# Implication of *Porphyromonas gingivalis* in colitis and homeostasis of intestinal epithelium

**DOI:** 10.1186/s42826-019-0029-6

**Published:** 2019-12-04

**Authors:** Yoojin Seo, Su-Jeong Oh, Ji-Su Ahn, Ye Young Shin, Ji Won Yang, Hyung-Sik Kim

**Affiliations:** 1 Department of Life Science in Dentistry, School of Dentistry, Pusan National University, Busandaehak-ro 49, Yangsan, 50612 Republic of Korea; 2 Dental and Life Science Institute, Pusan National University, Yangsan, 50612 Republic of Korea

**Keywords:** Periodontitis, *Porphyromonas gingivalis*, Colitis, Intestinal epithelium, Organoid

## Abstract

Emerging evidences have reported that periodontitis can be a risk factor for the pathogenesis of various systemic diseases. *Porphyromonas gingivalis* (*Pg*), one of the crucial pathogens in chronic periodontitis, has been spotlighted as a potential cause for the promotion and acceleration of periodontitis-associated systemic disorders. To investigate the association between *Pg* and intestinal disease or homeostasis, we treated *Pg*-derived lipopolysaccharide (LPS) in murine colitis model or intestinal organoid, respectively. *Pg*-derived LPS (*Pg* LPS) was administrated into chemically induced murine colitis model and disease symptoms were monitored compared with the infusion of LPS derived from *E. coli* (*Ec* LPS). Organoids isolated and cultured from mouse small intestine were treated with *Pg* or *Ec* LPS and further analyzed for the generation and composition of organoids. In vivo observations demonstrated that both *Pg* and *Ec* LPS exerted slight protective effects against murine colitis. *Pg* LPS did not affect the generation and growth of intestinal epithelial organoids. Among subtypes of epithelial cells, markers for stem cells, goblet cells or Paneth cells were changed in response to *Pg* LPS. Taken together, these results indicate that *Pg* LPS leads to partial improvement in colitis and that its treatment does not significantly affect the self-organization of intestinal organoids but may regulate the epithelial composition.

## Introduction

Periodontitis is a chronic oral inflammation which destroys the tooth-supporting periodontium including gingiva, alveolar bone and periodontal ligament [1]. This disease is one of the most prevalent chronic diseases. Over the decades, several bacteria have been reported to be associated with periodontitis [2]. In particular, *Porphyromonas gingivalis* (*Pg*) is considered as one of the crucial pathogenic bacteria for periodontitis [3–10]. A number of studies have proposed that *Pg* is involved in the onset and progression of extra-oral inflammatory disorders, such as diabetes, cardiovascular diseases, rheumatoid arthritis, adverse pregnancy outcomes and pulmonary diseases [11–15]. More recently, it has been demonstrated that periodontitis might be associated with inflammatory bowel diseases (IBD) [16, 17]. IBD describes a group of inflammatory conditions of the gastrointestinal tract and can be categorized into two major forms, Crohn’s disease and ulcerative colitis [18]. Although periodontitis and IBD might share similar etiologic pathways and large quantities of oral bacteria can reach the intestine through saliva flow [19], only few studies have experimentally reported the association between periodontitis-exacerbating pathogens and IBD [20, 21]. Therefore, in the present study, we sought to investigate the impact of *Pg* LPS in mouse colitis model induced by dextran sulfate sodium (DSS).

The epithelium of the digestive tract provides a large surface area for interaction of host and microbiota and serves as a primary balance system for tolerance and pathogen clearance [22]. The intestinal epithelium exhibits rapid turnover in 4–5 days and this robust regenerative capacity depends on stem cells residing in the bottom of crypts between villi [23]. Intestinal stem cells (ISCs) critically contribute to the homeostasis of gut epithelium by generating five epithelial cells, including enterocytes, goblet cells, Paneth cells, tuft cells and enteroendocrine cells. A number of studies have uncovered that stem cells from various tissues can directly recognize pathogen-associated molecular patterns (PAMPs) and their function including self-renewal and differentiation can be altered in response to PAMPs [24–27]. Given that oral cavity and gastrointestinal tract are directly linked and oral bacteria or their components can be frequently exposed to intestinal epithelium via saliva flow, we investigated the influence of *Pg* LPS on functions of ISCs. We utilized three-dimensional organoid culture models to assess the direct impact of *Pg* LPS on epithelial homeostasis or regeneration with the exception of other cell types in the intestine including immune cells or stromal cells which also respond to microbial components and subsequently regulate epithelial maintenance [28].

## Results

### *Pg LPS* exerts modest beneficial effects against DSS-induced colitis in mice

**Fig. 1:**
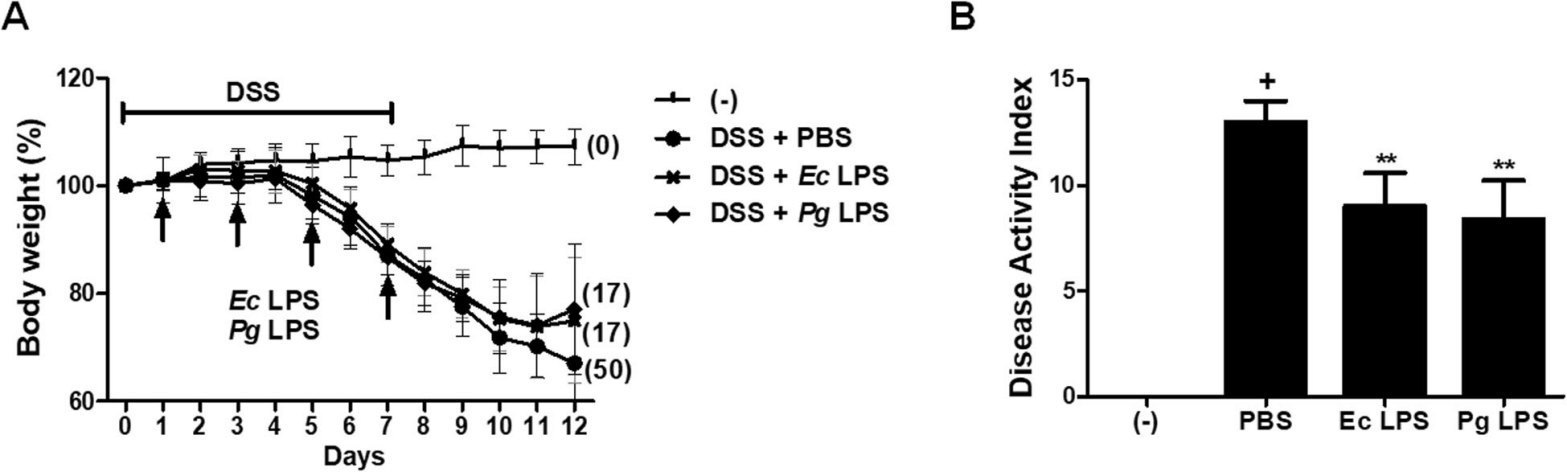
Monitoring of colitic mice. (**a**) Body weight loss and lethality of mice were monitored for 12 days. The body weight of each mouse at day 0 was calculated as 100%. Numbers in parentheses mean the percentage of dead mice. In ‘DSS + PBS’ group, 2 mice at day 11 and 1 mouse at day 12 died. In ‘DSS + *Ec*LPS’ and ‘DSS + *Ec*LPS’ group, 1 mouse at day12 died, respectively. (**b**) Disease activity index indicating colitis severity was scored at day 12. 6 mice per group were used. In (**b**), *p*-value significance was calculated by comparing other groups against (+) group (marked as +). **P < 0.01

**Fig. 2:**
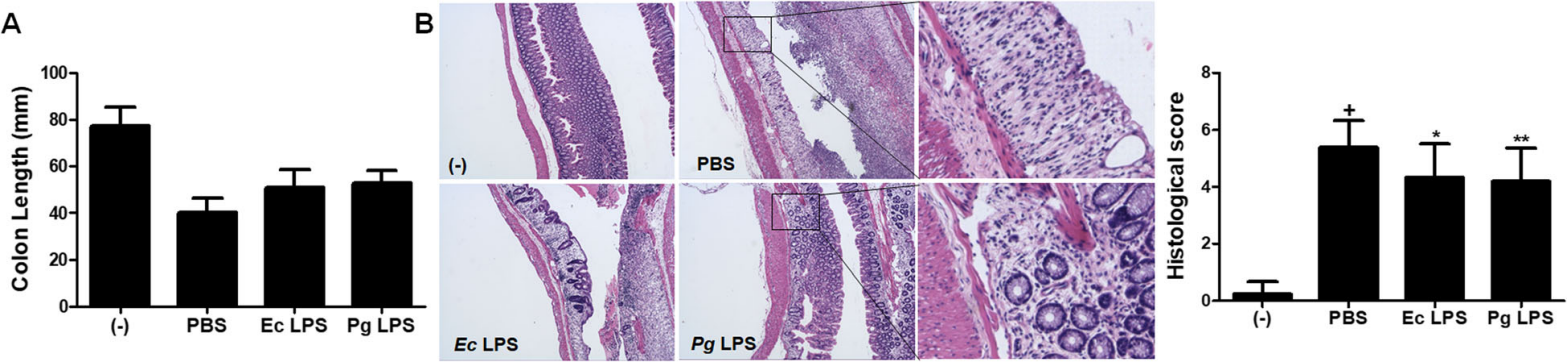
Gross and histological evaluation of colon. (**a**) Colon length was measured from proximal colon to rectum. (**b**) Destruction of epithelial structure and scattered infiltration of inflammatory cells were assessed as histopathological score in H&E stained section of colon tissues. Results are shown as mean ± SD. Representative areas showing the damaged area with typical structure destruction and leukocyte infiltration (black box) were magnified. In (**b**), p-value significance was calculated by comparing other groups against (+) group (marked as +). *P < 0.05, **P < 0.01

We first investigated whether the administration of *Pg* LPS can affect the severity of DSS-induced colitis. 3% DSS was added in drinking for 7 days to induce acute colitis and *Pg* LPS was orally infused at day 1, 3, 5 and 7. Interestingly, oral administration of *Pg* LPS or *Ec* LPS slightly ameliorated the loss of body weight and rescued mice from lethality compared to PBS-treated group (Fig. 1a). On day 12, the disease activity index (DAI) was significantly decreased in *Pg* or *Ec* LPS-treated group (Fig. 1b). After the assessment of DAI, the mice were sacrificed for gross and histological evaluation of colonic inflammation and damage. Colon length was reduced in PBS-treated group, however, it was moderately restored by *Pg or Ec* LPS treatment (Fig. 2a). H&E staining and histopathological analysis revealed that DSS treatment led to the destruction of epithelium and infiltration of inflammatory cells, and the administration of both *Pg* and *Ec* LPS significantly attenuated theses epithelial damages observed in colons of colitic mice (Fig. 2b). As expected from gross observations, the administration of both *Pg* and *Ec* LPS significantly attenuated the destruction of the epithelial structure, as well as the infiltration of lymphocytes (Fig. 2b). Taken together, our results suggest that *Pg* LPS exhibits moderate protective effects in chemically induced colitis of mice and its effect and potency are similar to *Ec* LPS.

### Pg LPS does not affect the morphology or growth of mouse intestinal organoids but regulates the expression of genes for epithelial cells

**Fig. 3:**
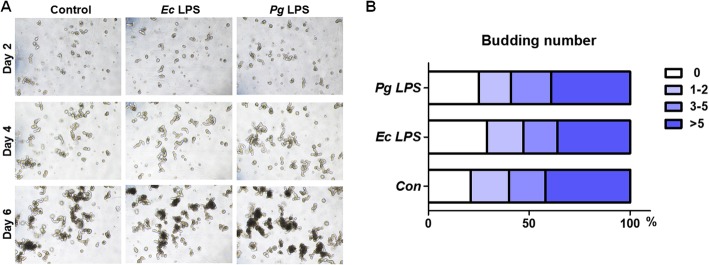
Observation of the organoid from mouse small intestine. The organoid was generated from crypt cells of moue small intestine and maintained using conditioned media (**a**) Photographs of organoids were taken at day 2, 4 and 6 in the presence or absence of LPS. (**b**) The number of budding was counted on day 5

**Fig. 4:**
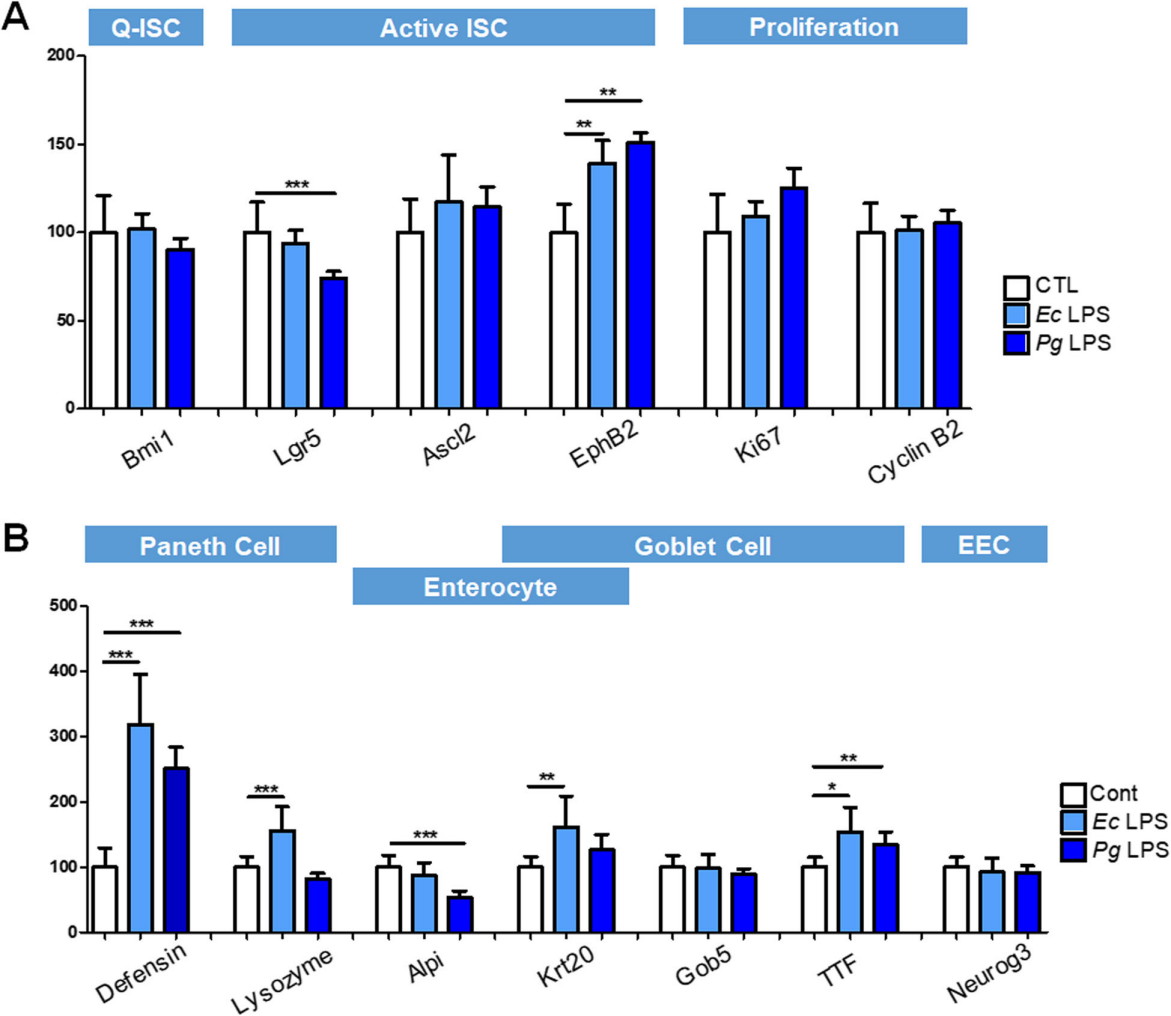
Quantification of markers for epithelial cell differentiation. The organoid was harvested at day 5 and selected markers for differentiation of epithelial cells were determined by qPCR. (**a**) Makers for stem cells and proliferation were measured. (**b**) Markers for Paneth cells, enterocytes, goblet cells and enteroendocrine cells were determined. Results are shown as mean ± SD. *P < 0.05, **P < 0.01, ***P < 0.001

In order to specifically analyze the impact of *Pg* LPS on intestinal epithelial cells, we generated 3D culture organoids from mouse small intestine and LPS was treated. Mouse small intestinal organoid (SIO) showed a spherical structure with appearance of surface protrusions as it grows (Fig. 3a). LPS from *Pg* or *Ec* did not alter the morphology of SIO nor the formation of budding structure (Fig. 3a and b). We then explored whether the differentiation of epithelial cells is regulated by *Pg* LPS by quantitative PCR of selected representative genes for each epithelial cell subtype. The expression of *Bmi1*, a marker for quiescent ISCs (Q-ISC), was not affected by *Pg* or *Ec* LPS treatment. Among markers of active stem cells, *Lgr5* expression was significantly down-regulated after *Pg* LPS treatment whereas *EphB2* expression was up-regulated (Fig. 4a). The expression of markers for the proliferation was not affected (Fig. 4a). *Pg* and *Ec* LPS treatment significantly elevated the expression of *Defensin*, a marker for Paneth cells, however, only *Ec* LPS significantly increased the expression of another Paneth cell marker, *Lysozyme* (Fig. 4b). One of markers for enterocytes, *Alpi* was reduced to a significant extent in response to *Pg* LPS (Fig. 4b). In addition, the expression of *TTF,* a goblet cell marker, was significantly up-regulated by LPS from both bacteria (Fig. 4b). Lastly, the differentiation of enteroendocrine cells (EECs) was not affected when determined by *Neurogenin3* expression (Fig. 4b). These results indicate that *Pg* LPS does not critically affect the morphology or growth of mouse SIO, but possibly regulates the differentiation of subtype cells which constitute intestinal epithelium based on the determination of changes in marker expression for stem cells, Paneth cells, enterocytes and goblet cells.

## Discussion

In the present study, we investigated the influence of LPS from *Pg*, a keystone bacterium in periodontitis, in intestinal inflammation and homeostasis. The inflammation initiated by periodontitis can lead to systemic inflammation, therefore, it is plausible to presume that periodontal diseases might exacerbate IBD symptoms. A study by Arimatsu et al. has demonstrated that oral administration of *Pg* changed the composition of gut microbiota, as well as the function of gut epithelial barrier [20]. Moreover, in the study, insulin resistance was elevated in *Pg*-fed mice. In another study, *Pg* administration damaged tight junction in small intestine and bacterial influx was followed. In addition, IL-6 level in small intestine and TNF-α level in large intestine were significantly elevated [21]. Recently, Blasco-Baque et al. established a murine model of periodontitis by infecting periodontium with *Pg*, *Fusobacterium nucleatum and Prevotella intermedia*. They observed modest or subtle changes of gut microbiota [29]. However, none of these studies investigated simple direct impact of *Pg* LPS in DSS-induced murine colitis model. In the present study, although we expected that the oral administration of *Pg* LPS might accelerate or aggravate colitis symptoms, however, on the contrary, *Pg* LPS treatment exerted modest protective effects against colitic mice. *Ec* LPS treatment showed similar effects. Saito et al. reported that the administration of *Ec* LPS significantly suppressed colonic inflammation induced by DSS. They also suggested that repeated stimulation with LPS reduced TNF-α production and up-regulated a negative regulator of Toll-like receptor (TLR) 4 signaling [30]. Along with this study, our present study proposes that *Pg* LPS might act through similar mechanisms or signaling as *Ec* LPS, at least in DSS-induced colitis. This observation supports the currently accepted theory that intestinal homeostasis is mainly maintained by TLR signaling, in spite of the continuous exposure to microbiota or their derivatives [31–33].

Among TLRs, the receptor for *Pg* LPS has been reported as TLR2, which is still controversial. Several studies have suggested that *Pg* LPS exhibits its activity via TLR2 [34, 35]. However, Ogawa et al. revealed that *Pg* LPS activates cells through TLR4 and not TLR2 by investigating the structure and function of lipid A of *Pg* LPS [36]. In addition, Hirschfeld et al. proposed that TLR2 activation of *Pg* LPS might be mediated by contaminated lipoprotein [37]. Therefore, it is still controversial through which *Pg* LPS exerts its activities. A recent study by Nativel et al. demonstrated that *Pg* LPS initiates pro-inflammatory signaling exclusively via TLR4 [38]. On the contrary, Andrukhov *etl al.* showed that *Pg* LPS and *Ec* LPS differently regulated the pattern of cytokine production from human gingival fibroblasts [39]. In the present study, we tried to investigate whether LPS from *Pg* or *Ec* differently regulates the function of intestinal stem cells using organoid culture system. Although the morphology, as well as the growth of the organoids were not affected by both LPS, *Pg* LPS exhibited different activities on the differentiation of epithelial cells compared with *Ec* LPS. Particularly, only *Pg* LPS down-regulated the expression of *Lgr5*, a representative marker for intestinal stem cells, and *Alpi*, a marker for absorptive enterocytes. Our finding is consistent with the results from Nalto et al. suggesting that LPS from different microbiota (*Acinetobater, Delftia* and *Stenotrophonas*) differently regulate the differentiation of epithelial cells by acting on Lgr5^+^ stem cells [40].

Our study has its limitation in that *Pg* LPS action in colitis was determined using only one chemically induced model. To provide convincing evidences for the association between periodontitis and IBD, future studies using a number of models including recently established CRISPR/Cas9-mediated knockout mice with intestinal phenotypes [41–43], might be required. In addition, single cell RNA sequencing for organoid analysis can provide more visualized trajectory exploration for epithelial differentiation.

## Conclusions

In conclusion, the present study indicates that LPS from periodontal pathogen, *Porphyromonas gingivalis*, exerts disease-ameliorating activities in chemically induced murine colitis model. We also found that *Porphyromonas gingivalis*-derived LPS differently regulates the lineage differentiation of intestinal epithelial cells compared to *E. coli*-derived LPS, whereas both LPS does not affect general growth and maturation of organoids mediated by intestinal stem cells.

## Materials and methods

### Colitis induction and evaluation

Animal experiments were conducted according to the approval and regulations of the Institute of Laboratory Animals Resources (PNU-2018-2034, Pusan National University). Experimental colitis was induced in mice by the addition of 3% (weight/volume) DSS (MP Biochemicals, Solon, OH) was added in drinking water for 7 days to induce the experimental colitis in C57BL/6 mice (male, 8–10 wks) [29]. Animals were divided into 4 groups as negative (normal drinking water, *n* = 6) control and vehicle (DSS + PBS, n = 6) controls and *Ec* LPS (10 μg/head, n = 6) and *Pg* LPS (10 μg/head, n = 6) treated group. LPS was administrated at day 1, 3, 5 and 7 per oral administration. Mice were monitored for 12 days for body weight and survival rate. On day 12, colitis severity of mice was measured by scoring disease activity index. All animals were sacrificed followed by the gross examination of colons and further histopathological evaluation using H&E staining.

### Colitis severity

Colitis severity was determined by scoring the disease activity index on stool consistency (graded from 0~4), rectal bleeding (0~4), coat roughness (0~4), and general activity (0~2).

### Histopathological examination

Colon samples were prepared, fixed in formalin, processed in cycles of alcohol-xylene changes, and embedded in paraffin. 5 μm sections of colon samples were prepared and stained with H&E. The infiltration of lymphocytes (graded from 0~4) and damages of intestinal epithelium (graded from 0~4 based on the measurement of damaged area) were graded blindly.

### Small intestinal organoid (SIO) culture

Murine small intestinal organoids were established with Intesticult organoid system (Stemcell Technologies, Vancouver, Canada) as manufacturer’s instruction with minor modification. Briefly, the proximal part (~ 10 cm) of duodenum was isolated and flushed with cold PBS 3 times. SI was then incised longitudinally and the intestinal villi and mucus were scraped off using a coverslip. Tissue was cut into 2–3 mm segments and collected into 50 ml tube containing cold PBS. The fragments were washed with vigorous shaking and settled down, followed by supernatant removal. This washing step was repeated until the supernatant was clear (usually 3–5 times). Crypts were detached using Gentle Cell Dissociation Reagent (Stemcell Technologies) by 20-min-long incubation at room temperature with gentle rocking (~ 20 rpm). After filtration with 70 μm cell strainer (BD Falcon, Franklin Lakes, NJ). Flow-through crypts were centrifuged at 290 g for 5 min. Pellets were resuspended in DMEM/F12 (Gibco, Grand island, NY) then centrifuged at 200 g for 5 min. Approximately 250 crypts were re-suspended in 20 μl of DMEM/F12 and 20 μl of Matrigel (Corning Life Sciences, Corning, NY) and total 40 μL of crypt-Matrigel suspension was seeded into each well of 24 well plate followed by incubation at 37 °C for 30 min. After polymerization of the Matrigel, 750 μl of IntestiCult™ Organoid Growth Medium (Stemcell Technologies) was added for culture maintenance with or without *Pg* or *Ec* LPS (10 μg/mL). At day 5, the budding number of organoids were calculated, followed by the harvesting and further lysis for RNA isolation.

### RNA isolation and quantitative real-time PCR (qPCR)

Total RNA of SIOs were extracted with RNeasy Mini Kit (Qiagen, Hilden, Germany) and cDNA was synthesized using ReverTra Ace® qPCR RT Master Mix (Toyobo, Osaka, Japan) as manufacturer’s instruction. The quantitative PCR (qPCR) was performed using SYBR Green reagents (Thermo Scientific) on a ABI 7500 real-time PCR instrument (Applied Biosystems, Carlsbad, CA). Primer sequences are summarized in Table 1. Relative expression levels of each marker were normalized to Gapdh expression.

**Table 1:** Primer sequences for qPCR

Gene	Forward primer	Reverse primer
Lgr5	GGGAGCGTTCACGGGCCTTC	GGTTGGCATCTAGGCGCAGGG
Bmi1	CCGGGATCTTTTATCAAGCA	TACCCTCCACACAGGACACA
Ascl2	CATGGAAGCACACCTTGACTG	CATCAAGCTTGCATTCAGCC
Ephb2	CAACGGTGTGATCCTGGACTAC	CACCTGGAAGACATAGATGGCG
Lysozyme	TGAACGTTGTGAGTTTGCCA	TGAGCTAAACACACCCAGTCG
TFF3	TAATGCTGTTGGTGGTCCTG	CAGCCACGGTTGTTACACTG
Alpi	CTGCCAAGAAGCTGCAGCCCA	GGCTAGGGGTGTCTCCGGTCC
Neurog3	GCATGCACAACCTCAACTC	TTTGTAAGTTTGGCGTCATC
Ki67	CCTTTGCTGTCCCCGAAGA	GGCTTCTCATCTGTTGCTTCCT
CyclinB2	GCCAAGAGCCATGTGACTATC	CAGAGCTGGTACTTTGGTGTTC
α-defensin-5	ACTGAGGAGCAGCCAGGGGA	ACGCGTTCTCTTCTTTTGCAGCC
Krt20	GGATTCGAGGTTCAAGTCACGG	TCTAGGTTGCGCTCCAGAGACT
Gob5	GGTGATCATCAGACCCCAGCATCAGT	AAGAGACTAAAACTGAGGAGCAGC

### Statistical analysis

The mean value of the different groups was demonstrated as the mean ± SD. All statistical comparisons were performed using one or two-way ANOVA followed by the Bonferroni post-hoc test for multi-group comparisons using the GraphPad Prism version 5.01 (GraphPad Software, San Diego, CA). Statistical significance designated as asterisks is indicated in the figure legends.

## Data Availability

All data produced or analyzed in the present study are available upon reasonable request.

## Abbreviations

DAI: disease activity index
DSS: dextran sulfate sodium
EEC: enteroendocrine cell
IBD: inflammatory bowel diseases
ISCs: intestinal stem cells
LPS: lipopolysaccharide
PAMPs: pathogen-associated molecular patterns
*Pg*: *Porphyromonas gingivalis*
Q-ISC: quiescent intestinal stem cell
SIO: small intestinal organoid
